# Serum IL-27 levels increase in subjects with hypothyroidism and are negatively correlated with the occurrence of nonalcoholic fatty liver disease

**DOI:** 10.3389/fendo.2023.1173826

**Published:** 2023-08-02

**Authors:** Yahui Wen, Heng Zhang, Ning Yang, Xia Gao, Zhe Chen, Jia Liu, Guang Wang

**Affiliations:** Department of Endocrinology, Beijing Chao-yang Hospital, Capital Medical University, Beijing, China

**Keywords:** hypothyroidism, IL-27, NAFLD, dyslipidemia, lipid metabolism

## Abstract

**Background:**

The level of serum interleukin-27 (IL-27) was significantly decreased in the obesity group. After injection of IL-27, obese mice showed significant weight loss,reduced fat accumulation, improved insulin resistance and hepatic steatosis.IL-27 plays a key role in the regulation of metabolic processes, but there are scarce data on circulating IL-27 levels in hypothyroidism. The purpose of this study was to assess the serum levels of IL-27 in patients with hypothyroidism and its relationship with NAFLD.

**Methods:**

185 participants were included in this cross-sectional survey. According to thyroid function, the subjects were classified into three groups: euthyroidism (n = 55), subclinical hypothyroidism (n = 53), and hypothyroidism (n = 77). Serum IL-27 concentrations were measured by ELISA.

**Results:**

Serum IL27 levels were significantly higher in subclinical hypothyroidism and hypothyroidism groups than in the euthyroidism group. Serum IL27 levels had a negative correlation with HOMA-IR,FBG,TG, subcutaneous fat,and visceral fat, and had a positive correlation with HDL-C (*P*< 0.05). Furthermore, logistic regression analysis indicated that IL-27 levels, HOMA-IR, and visceral fat showed significant associations with NAFLD after complete adjustment (*P*< 0.05). ROC curves showed that theoptimal cut-off value of serum IL-27 for discriminating NAFLD was 95.87pg/mL. The area under the ROC curve was 77.3% (95% CI = 0.694-0.851, p < 0.001).

**Conclusions:**

Serum IL-27 levels demonstrated a compensatory increase in patients with subclinical hypothyroidism or hypothyroidism and showed an independent association with NAFLD. Circulating IL-27 levels could predict the occurrence of NAFLD in hypothyroidism. These results suggested that altering the circulating levels of IL-27 may be a potential therapeutic target for NAFLD.

## Introduction

Hypothyroidism has an association with an increased risk of developing metabolic syndromecomponents, including obesity, insulin resistance and nonalcoholic fatty liver disease, which is widely prevalent in the common population (1). Clinical and subclinical hypothyroidism is an independent risk factor for NAFLD (2). The underlying mechanisms between NAFLD and hypothyroidism include oxidative stress, insulin resistance,dyslipidemia,metabolic syndrome, and direct action of TSH on hepatocytes (2, 3). Thyroid hormones play a key role in hepatic lipid metabolism, stimulating hepatic lipogenesis and causing hepatic fat accumulation through the thyroid hormone β receptor (4). Certain adipocytokines, such as interleukin-1, tumor necrosis factor-α (TNF-α), visfatin and leptin, and increased oxidative stress that occurs in some cases of hypothyroidism, may coincide with the development of insulin resistance (5). However,the biological mechanisms underlying the development and progression of NAFLD in patients with hypothyroidism are not fully understood (6).

NAFLD is a continuous process from simple steatosis to NASH and eventually to cirrhosis, where there is no effective treatment other than lifestyle modification and regular physical activity (7, 8). In overweight/obese NAFLD, a 7–10% weight loss is the target of most lifestyle interventions, and results in improvement of liver enzymes and histology. Dietary recommendations should consider energy restriction and exclusion of NAFLD-promoting components, macronutrient composition should be adjusted according to the Mediterranean diet, Both aerobic exercise and resistance training effectively reduce liver fat (9). Therefore, early identification of patients at a high risk of NAFLD is extremely important.Liver biopsy is the gold standard for the diagnosis of NADLD, but it has not been widely accepted due to its aggressiveness and high cost (10). At present,non invasive testing is used as a risk assessment tool for steatosis and fibrosis,clinicians mainly use ultrasound to detect fatty liver. Nevertheless, it depends on the experience and skill level of the operator (11). In addition, magnetic resonance spectroscopy (MRS) and magnetic resonance imaging (MRI) accurately determine the content of liver fat and the degree of fibrosis, but they have not been widely used in clinical practice due to the high cost (12). Therefore,noninvasive methods are urgently needed to identify NAFLD, monitor treatment effects, andtrack disease processes (13).

As a member of the IL-6/IL-12 cytokine family, IL-27 consists of two subunits, EBI3 and P28 (14, 15). IL-6 production has an association with decreased energy expenditure and increased body fat mass (16). The IL-27-IL-27Rα signaling pathway plays a key role in enhancing insulin resistance, promoting thermogenesis, and combating diet-induced obesity, which is a promising target for anti-obesity immunotherapy (17). IL-27 may be a novel target for the clinical treatment of metabolic diseases in the future. Currently, there are few inconsistent data on circulating IL-27 levels in patients with hypothyroidism. Hence, the purpose of this study was to assess the serum levels of IL-27 in patients with hypothyroidism and its relationship with NAFLD. Furthermore, the diagnostic accuracy of IL-27 was measured in the detection of NAFLD in hypothyroidism.

## Materials and methods

### Study group

A total of 185 patients (20-70 years) were included in this cross-sectional study. 77 patients were newly or previously diagnosed with clinical hypothyroidism, and 53 patients were newly or previously diagnosed with subclinical hypothyroidism, but had not received thyroid hormone replacement therapy within 3 months. 55 patients had normal thyroid functions. Exclusion criteria were: acute or chronic virus hepatitis, hepatolenticular degeneration, total parenteral nutrition, drug-induced liver disease, previous chronic heavy drinking, liver or kidney disease, heart failure,myocardial infarction breastfeeding or pregnancy, chronic inflammation, acute infection, and other specific diseases that cause fatty liver. Participants were recruited from the Endocrine Clinic at Beijing Chaoyang Hospital, Affiliated to Capital Medical University. All patients signed the informed consent, andthis study was approved by the Ethics Committee (2019-science-363) of Beijing Chaoyang Hospital, Capital Medical University.

### Biochemical and anthropometric measurements

Body weight, height, and waist were measured by trained personnel. Subcutaneous fat and visceral fat were measured by bioelectrical impedance analysis (OMRON, HDS-2000 DUALSCAN). Blood samples were taken from all subjects the next morning after fasting. BMI was calculated as weight(kg)/height(m)^2^. HDL-C, LDL-C, TG,TC, and FBG were tested using an autoanalyzer (Hitachi 747, Germany). Hemoglobin A1c (HbA1c) was detected using an HLC-723G7 analyzer (Tokyo, Japan) through high-performance liquid chromatography. Fasting insulin(FINS) was detected using chemiluminescence. Serum IL-27 levels were evaluated using an ELISA kit (R&D Systems, Minneapolis, MN, USA). Homeostasis model assessment–insulin resistance (HOMA-IR) was calculated as FINS (mIU/L) × FBG (mmol/L)/22.5 (18).

### Definition

According to thyroid function, all participants were classified into three groups: euthyroidism, subclinical hypothyroidism,TSH (>4.78uIU/mL) levels with normal FT4 and FT3 levels, and hypothyroidism, TSH (>4.78uIU/mL) levels with decreased FT4 levels. Reference values for thyroid function tests were as follows: TT4, 4.5-10.9ug/dL;FT4, 0.89-1.76ng/dL; TSH, 0.55-4.78uIU/mL.NAFLD was defined as no history of drinking or drinking< 210g/week (<140g/week for women), and ultrasound imaging outcomes were consistent with diffuse fatty liver disease (19).

### Statistical analysis

Data were analyzed with SPSS 26.0(IBM Corporation, New York) and GraphPad Prism9.0(Inc,CA,USA). The normal distribution of data was represented by the mean ± standard. Non-normally distributed data were represented bythe median and interquartile range. One-way ANOVA or Kruskal-Wallis test and Bonferroni *post hoc* test were used for comparison between groups. In addition, covariance (ANCOVA) and Bonferroni *post hoc* tests were used to compare the potential confounders of the adjusted differences between groups. The association between serum IL-27 levels and NAFLD was investigated using Pearson analysis. To further explore the association between serum IL-27 levels and dyslipidemia, linear regression was conducted.ROC curves were used to define the cut-off value of IL-27. The significance was indicated as P < 0.05, and all tests were two-tailed.

## Results

### Characteristics of study groups

**Figure 1:**
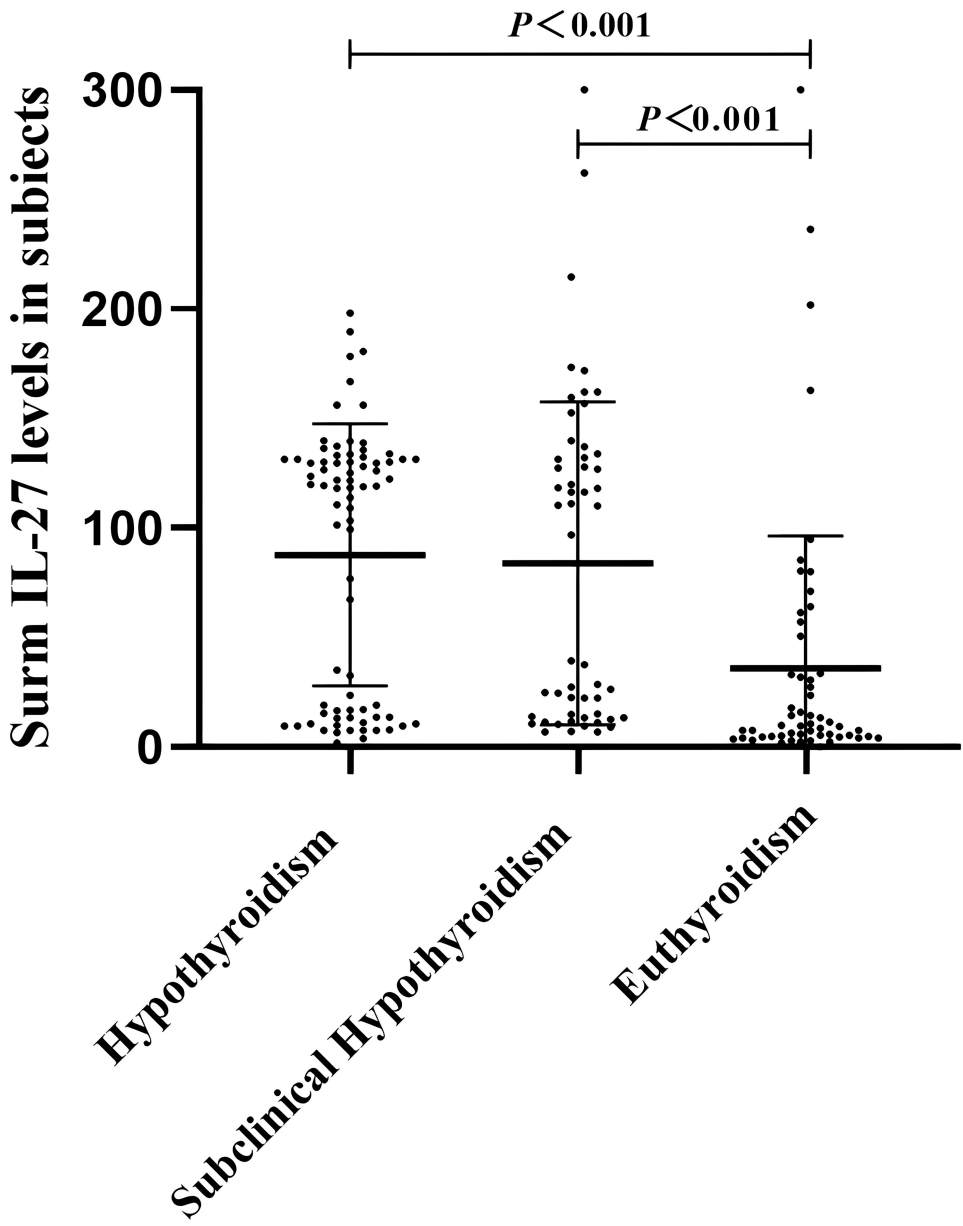
Comparison of IL-27 levels among the three groups.

The clinical characteristics of all the participants are shown in **Table 1**. The subjects were classified into three groups. Among the three groups, sex, age, ALT, AST, GGT, TG, LDL-C, HOMA-IR, fasting glucose, and subcutaneous fat showed no significant differences. Compared with the euthyroidism group,patients with hypothyroidism tended to have higher levels of BMI, TC, and visceral fat (P <0.05). The cytokine level of IL-27showed a higher serum concentration in patients with hypothyroidism compared to the euthyroidism group at 119.13(16.78,131.35) versus 8.94(4.65,32.07) pg/mL. Serum IL-27 levels were higher in the subclinical hypothyroidismgroup than in the euthyroidism group. There was no statistically significant difference in IL-27 between hypothyroidism and subclinical hypothyroidism patients (**Figure 1**).

**Table 1:** Clinical and biochemical characteristics of all the subjects.

Variable	Clinical Hypothyroidism n=77	Subclinical hypothyroidism n=53	Euthyroidism n=55	*P*
Sex,male,n(%)	17 (22.1)	7 (13.2)	7 (12.7)	0.262
Age,years	45.49 ± 11.78	43.62 ± 12.43	41.29 ± 8.64*	0.105
BMI,kg/m2	25.09 ± 4.31	23.99 ± 4.03	23.36 ± 3.09*	**0.040**
AST,U/L	23.0 (18.0,29.0)	21.0 (18.0,26.0)	20 (17.75,23.67)	0.078
ALT,U/L	20.0 (13.0,32.0)	18.0 (13.0,29.0)	16.0 (12.0,24.0)	0.115
GGT,U/L	19.0 (12.0,25.6)	17.0 (11.6,24.5)	18.0 (12.0,25.5)	0.436
TG,mmol/L	1.48 (0.81,1.84)	1.42 (0.95,2.04)	1.08 (0.91,1.44)	0.162
TC,mmol/L	5.38 (4.78,6.30)	4.87 (4.22,6.00)*	4.92 (4.23,5.58)**	**0.013**
LDL-C,mmol/L	3.12 (2.69,4.30)	2.88 (2.49,4.3)	2.92 (2.47,3.57)	0.172
FBG,mmol/L	4.57 (4.29,5.14)	4.81 (4.26,5.13)	4.56 (4.24,4.83)	0.220
HOMA-IR	1.73 (1.21,2.16)	1.82 (1.39,2.64)	1.76 (1.38,2.19)	0.298
sTSH,uIU/ml	55.92 (9.4,105.2)	6.5 (5.5,10.5)***	2.3 (1.6,2.9)***,^###^	**<0.001**
Visceral fat,cm^2^	75.0 (43.0,92.0)	66 (41.0,94.0)	62.0 (40.5,76.0)*	**0.017**
Subcutaneous fat,cm^2^	186.0 (144.0,241.0)	192.0 (166.0,245.0)	184.5 (124.75.249.25)	0.190
IL-27,pg/mL	119.13 (16.78,131.35)	96.78 (13.87,132.00)	8.94 (4.65,32.07)***,^###^	**<0.001**

Data were presented as the mean ± SD or median (interquartile range). P values for categorical variables were calculated using Chi-square test, and P values for continuous variables were calculated using one-way ANOVA test or Kruskal–Wallis test with the Bonferroni post hoc test. Bold indicates P value < 0.05. *Compared with hypothyroidism. *P < 0.05; **P < 0.01; ***,### P < 0.001. BMI, body mass index; AST, aspartate tansaminase; ALT, alanine transaminase; GGT, gamma glufamyl transferase; TG, triglycerides; TC, total cholesterol; HDL-C, high-density lipoprotein cholesterol; LDLC, low-density lipoprotein cholestero; FBG, fasting blood glucose; HOMA-IR, homeostasis model assessment -insulin resistance.

### Correlation between clinical parameters and serum IL-27 levels in hypothyroidism

**Figure 2:**
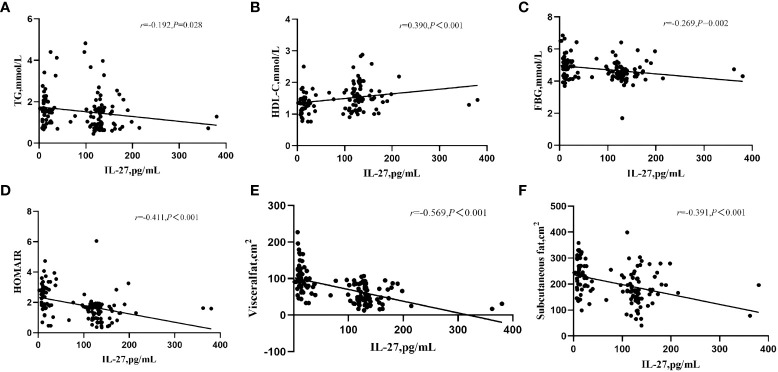
Correlation of serum IL-27 levels with TF **(A)** HDL-C **(B)**, FBG **(C)**, HOMIR **(D)**, visceral fat **(E)**, and subcutaneous fat **(F)** in Hypothyroidism. *P* value <0.05 were considered statistically significant.

Correlation analysis was used to investigate the association between serum IL-27 levels and metabolic parameters in patients with hypothyroidism (**Figure 2**). The concentration of circulating IL-27 was negatively related to TG(r=-0.192,P=0.028), FBG(r=-0.269, P=0.002), subcutaneous fatr=-0.391, P<0.001), visceral fat(r=-0.569, P<0.001), HOMA-IR(r=-0.411, P<0.001), and was positively related to HDL-C(r=0.39, P<0.001).

### Independent relationship between serum IL-27 levels and NAFLD in hypothyroidism

According to the diagnosis of NAFLD, patients with hypothyroidism or subclinical hypothyroidism were classified into two groups.There was no significant difference in TC, LDL-C, sTSH, FT3, and FT4 between the two groups. Patients with NAFLD had higher age, BMI, AST, ALT, GGT, TG, FBG, HOMA-IR, subcutaneous fat, visceral fat and lower HDL-C,IL-27 compared to those without NAFLD(**Table 2**). To identify whether serum IL-27 levels had an independent correlation with NAFLD, logistic regression analysis was performed among all participants (**Table 3**). The results indicated that lower serum IL-27 levels had a significant association with NAFLD [OR (95%CI), 0.97 (0.96-0.99)](P < 0.001). In addition,higher HOMA-IR[OR (95%CI), 16.85(2.18,130.45)] and visceral fat[OR (95%CI), 1.10(1.01,1.19)](P < 0.05) also had an independent association with NAFLD.

**Table 2:** Comparison of main metabolic related indexes between NAFLD and non-NAFLD patients with hypothyroidism.

Variable	NAFLD n=48	non-NAFLD n=82	*P*
Sex,male,n(%)	14 (29.16)	10 (12.19)	**0.016**
Age,years	49.06 ± 11.88	42.19 ± 11.45	**0.001**
BMI,kg/m^2^	27.94 ± 3.97	22.70 ± 2.99	**<0.001**
AST,U/L	24.0 (20.2,28.0)	21.0 (17.0,26.5)	**0.060**
ALT,U/L	22.0 (16.0,32.5)	17.0 (12.0,28.0)	**0.028**
GGT,U/L	24.0 (16.5,34.5)	14.0 (11.0.19.0)	**<0.001**
TG,mmol/L	1.67 (1.32,2.27)	1.05 (0.74,1.62)	**<0.001**
TC,mmol/L	5.25 (4.22,6.46)	5.10 (4.54,6.21)	0.622
HDL-C,mmol/L	1.25 (1.03,1.44)	1.51 (1.30,1.79)	**<0.001**
LDL-C,mmol/L	3.59 ± 1.23	3.24 ± 1.06	0.106
FBG,mmol/L	5.05 ± 0.75	4.57 ± 0.63	**<0.001**
HOMA-IR	2.77 (1.82,3.18)	1.59 (0.93,1.81)	**<0.001**
Subcutaneous fat,cm^2^	243.53 ± 59.22	178.93 ± 62.75	**<0.001**
Visceral fat,cm^2^	96.0 (83.5,124.0)	48.0 (31.5,76.5)	**<0.001**
sTSH,uIU/mL	11.53 (6.34,63.28)	10.58 (6.11,72.71)	0.952
FT3,pg/mL	2.90 (2.35,3.38)	2.87 (2.23,3.26)	0.337
FT4,ng/dL	0.91 (0.60,1.24)	0.95 (0.57,1.15)	0.497
IL-27,pg/mL	13.74 (10.01,24.11)	126.82 (110.77,136.93)	**<0.001**

Data were presented as the mean ± SD or median (interquartile range). P values for categorical variables were calculated using Chi-square test, and P values for continuous variables were calculated using one-way ANOVA test or Kruskal–Wallis test with the Bonferroni post hoc test. Bold indicates P value < 0.05. BMI, body mass index; AST, aspartate tansaminase; ALT, alanine transaminase; GGT, gamma glufamyl transferase; TG, triglycerides; TC, total cholesterol; HDL-C, high-density lipoprotein cholesterol; LDLC, low-density lipoprotein cholestero; FBG, fasting blood glucose; HOMA-IR, homeostasis model assessment -insulin resistance.

**Table 3:** Correlation analysis between serum IL27 concentration and non-alcoholic fatty liver disease in hypothyroidism.

Variable	Single-factor analysis		multivariate analysis	
	OR (95%CI)	*P*	OR (95%CI)	*P*
Age,years BMI,kg/m^2^	1.02 (0.96,1.07) 1.08 (1.04,1.13)	0.604 **0.001**	0.91 (0.62,1.33)	0.617
AST,U/L	1.02 (0.99,1.07)	0.166		
ALT,U/L	1.04 (1.00,1.08)	**0.031**	1.03 (0.96,1.12)	0.352
GGT,U/L	1.01 (0.99,1.02)	0.348		
TG,mmol/L	2.13 (1.16,3.93)	**0.015**	2.34 (0.81,6.79)	0.116
TC,mmol/L	1.32 (0.89,1.96)	0.166		
HDL-C,mmol/L	0.15 (0.02,0.87)	**0.035**	3.15 (0.16,61.03)	0.448
LDL-C,mmol/L	1.41 (0.85,2.31)	0.181		
HOMA-IR	8.37 (2.67,26.22)	**<0.001**	16.85 (2.18,130.45)	**0.007**
Subcutaneous fat,cm^2^	1.01 (0.99,1.02)	**0.012**	1.01 (0.99,1.03)	0.248
Visceral fat,cm^2^	1.08 (1.00,1.02)	**<0.001**	1.10 (1.01,1.19)	**0.027**
sTSH,uIU/mL	1.01 (0.99,1.02)	0.350		
FT3,pg/mL	1.02 (0.53,1.94)	0.961		
FT4,ng/dL	0.82 (0.23,2.93)	0.756		
IL-27,pg/mL	0.95 (0.93,0.96)	**<0.001**	0.97 (0.96,0.99)	**0.001**

Bold indicates P value < 0.05.

**Figure 3:**
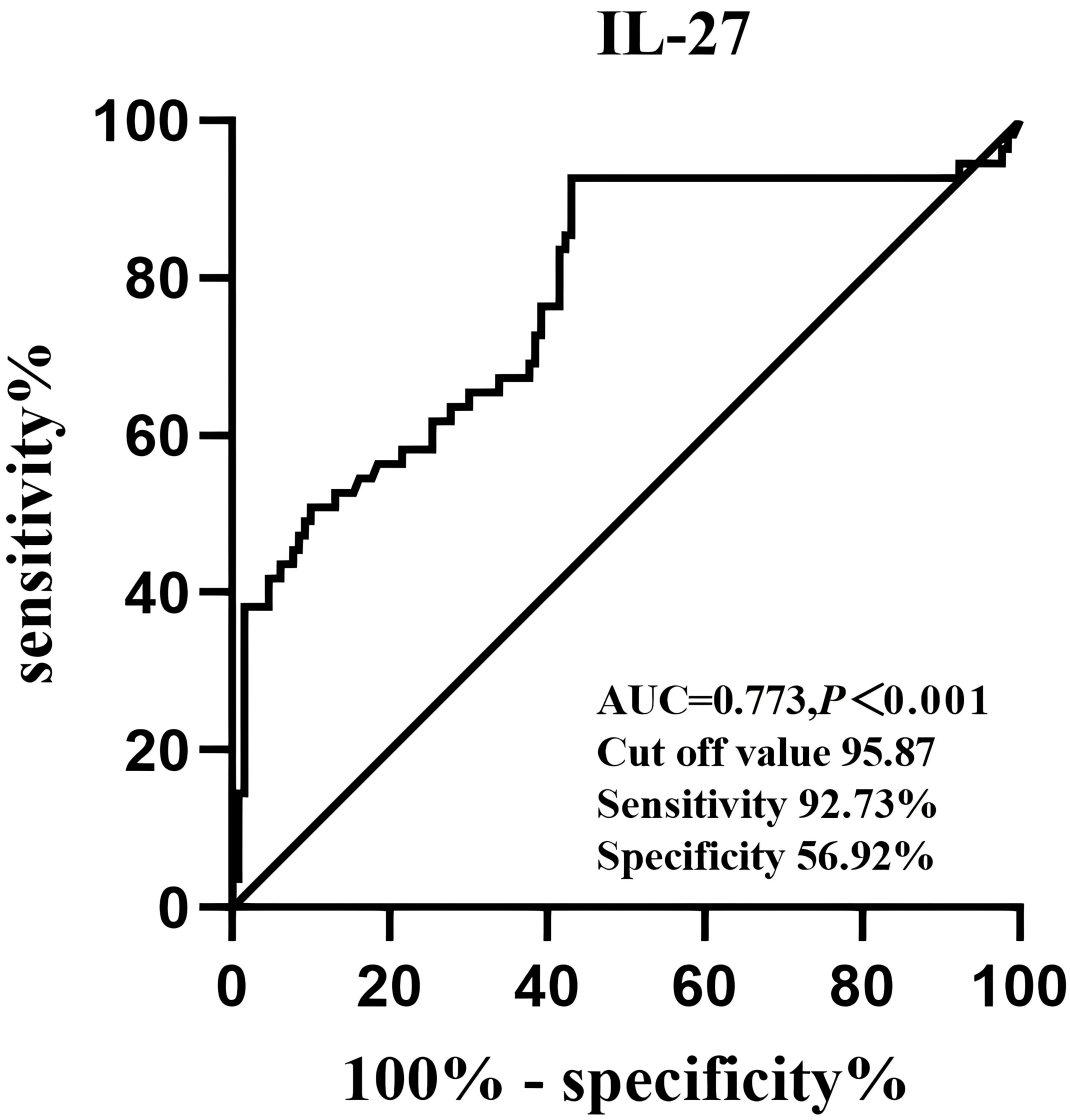
Receiver operative characteristics curves and cut off values of serum IL-27 levels for NAFLD.

Finally, the diagnostic value of IL-27 for NAFLD was analyzed by ROC curves (**Figure 3**). The optimal cut-off value of serum IL-27 for discrimination of NAFLD was 95.87pg/mL (AUC = 0.773, P < 0.001).

## Discussion

In this study, a compensatory increase in the concentration of serum IL-27 was observed in patients with hypothyroidism or subclinical hypothyroidism. Serum IL-27 levels were negatively associated with FBG, HOMAIR, subcutaneous fat, visceral fat, and TG, and positively associated with HDL-C in patients with hypothyroidism. More notably, lower circulating IL-27 levels in patients with hypothyroidism were independently related to NAFLD. These results suggested that IL-27 could be a promising therapeutic target for NAFLD in patients with hypothyroidism.

Hypothyroidism is a common endocrine disorder characterized by increased sensitivity to cold and unexpected weight gain, suggesting a change in response to cold and energy expenditure (20). Thyroid hormones play an important role in the normal function and cold-induced thermogenesis of brown adipose tissues in rodents (21, 22). Wang et al. reported that IL-27Rα-deficient mice were cold-intolerant because of impaired adaptive thermogenesis, and IL-27 improved thermogenesis and directly targeted adipocytes to counteract obesity (17). Other studies have shown that IL-27 exerts its beneficial effects by the upregulation of adaptive thermogenesis in brown adipose tissues (23). Consistent with their findings, a compensatory increase in the concentration of serum IL-27 was observed in patients with hypothyroidism or subclinical hypothyroidism. IL-27 may combat hypothermia in patients with hypothyroidism through its febrile effects.

NAFLD is a worldwide health problem and has an increased risk of diabetes, CVD and CKD (24). NAFLD is a complex and heterogeneous disease that is inaccurately diagnosed by liver biopsy (25). Current therapeutic strategies mainly focus on lifestyle interventions, and there are still limited appropriate drugs specifically available for NAFLD (26). Therefore, the discovery of non-invasive detection, novel therapeutic targets and strategies shall be required. Patients with hypothyroidism are at an increased risk of NAFLD. Hypothyroidism plays an important role in the development and progression of NAFLD (27).

Dyslipidemia related to hypothyroidism leads to intrahepatic fat accumulation, resulting in NAFLD and thus leading to the development of hepatic insulin resistance (1). Thyroid hormones increase the expression of HMG-CoA reductase in the liver to increase cholesterol synthesis (28). Plasma cholesteryl ester transfer proteins (CETPs) are decreased in a hypothyroid state, which shift cholesterol from HDL-C to LDL-C and VLDL (29). Consistently, data showed that NAFLD patients with hypothyroidism had higher BMI, LDL-C, FBG, HOMA-IR, subcutaneous fat, visceral fat, and lower HLD-C than non-NAFLD patients. It was found that IL-27 exerted a protective effect against diabetes by ameliorating STZ-induced hyperglycemia and islet inflammation. The hypertrophy of chronic inflammation and white adipocytes in white adipose tissues was blocked and HFD-induced liver steatosis was suppressed by Il-27 gene transfer (23). This study showed that serum IL-27 levels were negatively associated with FBG, HOMAIR, subcutaneous fat, visceral fat, and TG, and positively associated with HDL-C. Therefore, the findings support previous observations that IL-27 could improve insulin resistance and reduce fat accumulation. Meanwhile, data suggested that IL-27 could be involved in lipid metabolism. Thus, it was hypothesized that IL-27 may improve fatty liver by improving insulin resistance and reducing blood lipids. Besides, NAFLD patients with hypothyroidism had lower IL-27 compared to those without NAFLD. It can be speculated that the increase of IL-27 content may improve NAFLD. IL-27 levels may be a potential therapeutic target for dyslipidemia and NAFLD. The role of circulating IL-27 in improving NAFLD in hypothyroidism needs further investigation.

Multiple studies have demonstrated variables for predicting NAFLD (30). The noninvasive screening mode facilitates the timely identification and intervention of NAFLD and its complications in high-risk patients (31). Baseline HOMA-IR and weight gain were used as predictors of NAFLD incidence in a 7-year prospective study (32). Logistic regression analysis displayed that circulating IL-27 levels,HOMA-IR, and visceral fat were independently related to an increased risk of NAFLD. It was demonstrated that IL-27 could also predict the occurrence of NAFLD. In this study,IL-27 could predict NAFLD with an AUC of 0.897 (95% CI 0.757-0.859). In conclusion, the exact mechanism by which IL-27 regulates NAFLD metabolism needs to be further studied.

This study had a number of limitations.First, the sample size was small and the cross-sectional study failed to construct a causal relationship between IL-27 and disease. Second, other potential confounders were not eliminated, particularly cold exposure and exercise. Finally, only the Chinese population was included in this study, thus making the generalizability of the findings an issue.

In summary, serum IL-27 levels had a compensatory increase in patients with hypothyroidism or subclinical hypothyroidism and had an independent association with NAFLD. This study manifests that altering circulating IL-27 levels could predict the occurrence of NAFLD in hypothyroidism.

## Data availability statement

The original contributions presented in the study are included in the article/**Supplementary Material**. Further inquiries can be directed to the corresponding author.

## Ethics statement

The studies involving human participants were reviewed and approved by Ethics Committee of Beijing Chaoyang Hospital, Capital Medical University. The patients/participants provided their written informed consent to participate in this study.

## Author contributions

All authors contributed to the study conception and design. Material preparation, data collection and analysis were performed by YW, HZ, XG, NY, and ZC. The first draft of the manuscript was written by YW. The paper was revised by JL and GW. All authors contributed to the article and approved the submitted version.
